# Genetic diversity of *Nyssorhynchus* (*Anopheles*) *darlingi* related to biting behavior in western Amazon

**DOI:** 10.1186/s13071-019-3498-4

**Published:** 2019-05-17

**Authors:** Melina Campos, Diego Peres Alonso, Jan E. Conn, Joseph M. Vinetz, Kevin J. Emerson, Paulo Eduardo Martins Ribolla

**Affiliations:** 1 Biotechnology Institute (IBTEC) & Biosciences Institute at Botucatu (IBB), Sao Paulo State University (UNESP), Sao Paulo, Brazil; 2 Department of Biomedical Sciences, School of Public Health, University at Albany (State University of New York), Albany, NY USA; 3 Wadsworth Center, New York State Department of Health, Albany, NY USA; 4 Division of Infectious Diseases, Department of Medicine, University of California, San Diego, La Jolla, California USA; 5 Laboratorio de Investigación y Desarrollo, Departamento de Ciencias Celulares y Moleculares, Instituto de Medicina Tropical “Alexander von Humboldt”, Universidad Peruana Cayetano Heredia, Lima, Peru; 6 Biology Department, St. Mary’s College of Maryland, St. Mary’s City, MD USA

**Keywords:** *Nyssorhynchus* (*Anopheles*) *darlingi*, Biting behavior, Genetic population, Individual mosquito scale, Malaria vector, SNPs, Genetic heterogeneity

## Abstract

**Background:**

In the Amazon Basin, *Nyssorhynchus* (*Anopheles*) *darlingi* is the most aggressive and effective malaria vector. In endemic areas, behavioral aspects of anopheline vectors such as host preference, biting time and resting location post blood meal have a key impact on malaria transmission dynamics and vector control interventions. *Nyssorhynchus darlingi* presents a range of feeding and resting behaviors throughout its broad distribution.

**Methods:**

To investigate the genetic diversity related to biting behavior, we collected host-seeking *Ny. darlingi* in two settlement types in Acre, Brazil: Granada (~ 20-year-old, more established, better access by road, few malaria cases) and Remansinho (~ 8-year-old, active logging, poor road access, high numbers malaria cases). Mosquitoes were classified by the location of collection (indoors or outdoors) and time (dusk or dawn).

**Results:**

Genome-wide SNPs, used to assess the degree of genetic divergence and population structure, identified non-random distributions of individuals in the PCA for both location and time analyses. Although genetic diversity related to behavior was confirmed by non-model-based analyses and *F*_*ST*_ values, model-based STRUCTURE detected considerable admixture of these populations.

**Conclusions:**

To our knowledge, this is the first study to detect genetic markers associated with biting behavior in *Ny. darlingi*. Additional ecological and genomic studies may help to understand the genetic basis of mosquito behavior and address appropriate surveillance and vector control.

**Electronic supplementary material:**

The online version of this article (10.1186/s13071-019-3498-4) contains supplementary material, which is available to authorized users.

## Introduction

*Nyssorhynchus* (*Anopheles*) *darlingi* [1] is the main Neotropical malaria vector in the Amazon Basin due to its role in human *Plasmodium* transmission [2–4]. Following a decrease in the number of malaria cases in the Americas from 2005 to 2014, an increase was observed in the following three years, with Brazil and Venezuela as the countries contributing the largest number of cases [5, 6]. In Brazil, transmission remains entrenched in the Amazon Basin, which accounts for 99.5% of the country’s malaria burden [7]. Human migration to and within this region over the past century has been accompanied by dramatic environmental modification and the promotion of malaria transmission [8–11]. Deforestation and anthropogenic land changes are known to be associated with increases in *Ny. darlingi* presence and abundance and thus malaria risk [12–14].

Root [15] first described *Ny. darlingi* based on morphological characters of the egg, fourth-instar larva, pupa, male and female adults. Thenceforth, successive studies reported morphological, behavioral, ecological and genetic heterogeneity throughout its broad distribution from Central to South America [16–19]. Nevertheless, *Ny. darlingi* had been considered to be a monophyletic species until Emerson et al. [20] proposed the occurrence of three putative species based on well-supported genetic clusters. In their study, Neotropical biogeographical events explained dispersal of *Ny. darlingi* populations, at a continental scale, leading to diversification within this species. However, additional genetic diversity at a local scale has been demonstrated for this vector based on ecological differences [21, 22] and seasonality [23].

At least three aspects of mosquito behavior are especially important in determining pathogen transmission rates to humans: anthropophily, endophagy and endophily. The first is the predilection of a vector to blood-feed on humans instead of other animals, the second is a preference for biting inside houses, and the last is indoor resting behavior after a blood meal. These traits are known to vary within and between Anophelinae mosquito species that transmit malaria [24]. *Nyssorhynchus darlingi* demonstrates high anthropophilic behavior in many areas, sometimes combined with opportunistic zoophilic (non-human) feeding [25, 26]. Depending on location, house type, and host availability, among other environmental factors, it can display exophily/exophagy [27, 28] or endophily/endophagy [29] behavior or both [4, 30, 31]. Variation in peak biting times and patterns is also high [32, 33].

Heterogeneity of vector behavior could be the result of genetically differentiated subpopulations or behavioral plasticity, i.e. individuals with the same genotype adopt different behaviors. Behavioral plasticity seems to be the more likely explanation in malaria vectors. For example, studies on *Anopheles farauti* in the Solomon Islands identified a single population with individuals which fed indoors and outdoors, and early in the evening as well as late at night [34]. Furthermore, a study of biting time (early evening, dawn) and location (indoor, outdoor) in *An. arabiensis* analyzed 34 coding SNPs in 8 clock genes, but found no linkage between these phenotypes and the candidate clock genes known to influence behavior in other Diptera [35].

With these findings in mind, and a general dearth of such studies in Neotropical malaria vectors, the present article investigated the extent of population genetic diversity in *Ny. darlingi* on a microgeographical scale and its association with biting behavior. We tested the hypothesis that there was population genetic structure in this species associated with blood-feeding location (indoors or outdoors) and time (dusk or dawn) using genome-wide SNPs. Understanding the genetic contribution to mosquito biting behavior could lead to the development of molecular tools for more precise vector surveillance and malaria control.

## Methods

### Samples

**Fig. 1:**
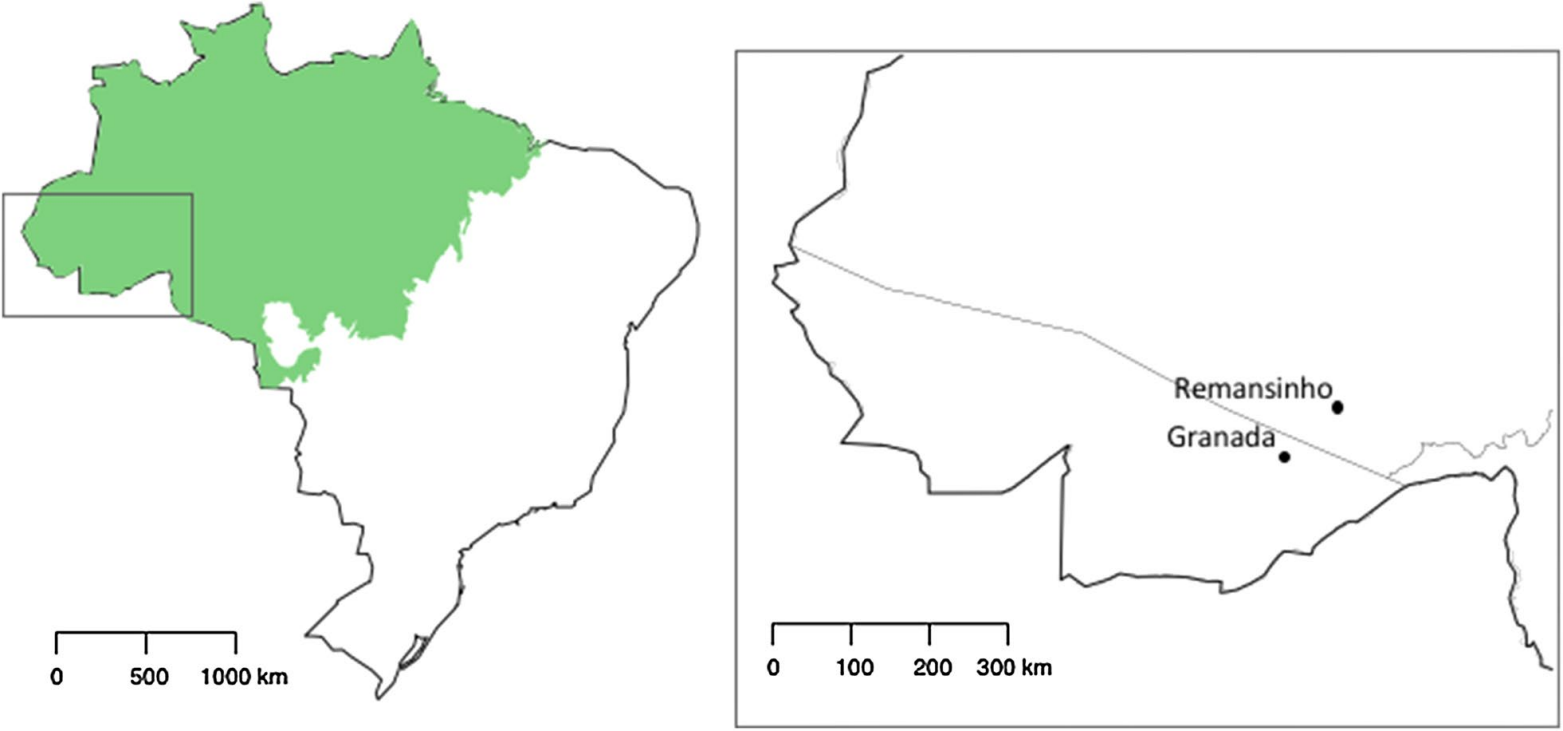
Map of Brazil showing collection points in Brazilian Amazon Basin. Square box: zoom in showing Acre and Amazonas states and the two collection localities, Granada and Remansinho

Collections of *Nyssorhynchus darlingi* adults were performed outdoors (peridomestic, within 10 m of each house) and indoors in two rural settlements Acre State in April 2011, the older more settled Granada (9°45′S, 67°04′W) and the newer, more recently deforested Remansinho (9°29′S, 66°34′W) (Fig. 1). Collections were performed using human landing catch (HLC) by the authors MC and PEMR, between 18:00–6:00 h. All collected specimens were identified using the key of Consoli & Lourenço-Oliveira [36] based on morphological characters and conserved individually in Eppendorf tubes at – 20 °C; only *Ny. darlingi* specimens were used for further analysis.

### SNP genotyping

DNA was individually extracted using ReliaPrep™ Blood gDNA kit (Promega, Madison, USA) and DNA concentration was estimated using a Qubit fluorometer (Invitrogen, Carlsbad, USA). Double restriction digestion of 200 ng of high-quality genomic DNA with *EcoR*I-*Msp*I restriction enzymes was performed in a 40 µl reaction volume and then purified with AMPure XP beads following the manufacturer’s protocol (Beckman Coulter, California, USA). Hybridized customized adapters P1 (0.3 µM) and P2 (4.8 µM) were ligated to the digested DNA (T4 DNA Ligase, Promega) as described in Campos et al. [21]. After another purification with AMPure XP beads, DNA was size selected on an agarose gel to 350–400 bp followed by another AMPure XP bead purification. PCR amplification for Nextera® indexing was carried out to generate Illumina sequencing libraries, according to these cycling conditions: an initial denaturation step at 72 °C for 3 min and at 95 °C for 30 s, followed by 16 cycles of 95 °C for 10 s, annealing at 55 °C for 30 s, elongation at 72 °C for 30 s, and a final extension cycle at 72 °C for 5 min, then each PCR product was purified one last time. Samples were individually quantified using the KAPA library quantification kit (KAPA Biosystems, Wilmington, USA) and equimolarly combined to compose a final library. Final libraries were quantified, normalized, denatured, and finally loaded on the Illumina reagent cartridge 150-cycle of paired-end sequencing in a Hiseq 2500 (Genomics and Bioinformatics Core, State University of New York at Buffalo).

### SNP data processing

Raw ddRAD reads were processed to identify SNP loci within and between individuals using scripts implemented in Stacks v1.31 [37]. First, sequences were quality filtered using the default parameters of *process_radtags* script. Then, each individual’s sequence reads were aligned to the *Ny. darlingi* reference genome [38] using Bowtie2 with default parameters [39]. Aligned reads were input to *ref_map.pl* Perl script in Stacks, using a minimum of 5 reads (*−m*) to report a stack and join the catalog. The dataset was corrected using another Stacks script called *rxstacks* with the following parameters: prune out non-biological haplotypes unlikely to occur in the population (*−prune_haplo*), minimum log-likelihood required was −10.0 (*−lnl_lim*). A new catalog was built by *cstacks* Stacks’ script and each individual was matched against the catalog with *sstacks* script. We then used the *populations* script in Stacks to filter loci that were called in at least 50% (*−r*) of all samples, i.e. the first step was performed without population information to avoid population bias in the SNP selection. This latter step was run with minimum coverage of 5 (−m) and a random single SNP for each RAD locus was selected (*write_random_snp*). The *populations* script produced genotype output in several formats (e.g. VCF, Genepop) and summary statistics such as nucleotide diversity, pairwise *F*_*ST*_ and private alleles.

### SNP data analysis

Principal components analysis (PCA) and discriminant analysis of principal components (DAPC) were performed in the R package *Adegenet* [40]. The former described global diversity overlooking group information whereas DAPC maximizes differences between groups and minimizes variation within clusters. The optimum number of genetic clusters in DAPC was the lowest Bayesian information criterion (BIC) associated with several *K*-means calculated. Number of retained PCs for DAPC analysis was calculated as described [41]. For model-based method, we used Bayesian clustering algorithm implemented in STRUCTURE (Pritchard et al., 2000), which was run 40 times for each *K* value that ranged from 1 to 6. STRUCTURE was run with admixture model and correlated allele frequencies, ‛burn-inʼ of 100,000 generations and MCMC chain of 1,000,000 generations. The Evanno method [42] was used to determine the optimal value of *K* for runs implemented in structureHarvester [43].

## Results

### SNP genotyping

A total of 128 individual mosquitoes were sequenced from both settlements, Granada (*n* = 96) and Remansinho (*n* = 32). We considered four collection categories: indoor collection at dusk, i.e. 18:00–21:00 h; indoors at dawn, i.e. 3:00–6:00 h; outdoors at dusk and outdoors at dawn (Table 1). Overall collections showed a preference for outdoor biting behavior and a peak of mosquito density between 19:00–21:00 h. From 124,841,110 ddRAD tag sequences, ~ 104 million sequences passed several levels of quality filtering and 34.9% (± 9.8 SD) of this set of reads was aligned to the *Ny. darlingi* genome [38]. An average (± SD) of 10,107 ± 4,123 ddRAD loci were genotyped per sample, and 25% presented SNPs (Additional file 1: Table S1). Analyses included endophagic (indoor) and exophagic (outdoor) specimens collected at dusk from both settlements, whereas, from Granada, analyses included the dusk and dawn collections, since the number of specimens collected at dawn in Remansinho were very low and were not included in this study. The number of SNPs included in each analysis was determined by including only those loci that were scored in > 50% of all individuals included for that particular analysis. Population assignment was performed after the SNPs data set selection.

**Table 1:** Number of *Nyssorhynchus darlingi* genotyped by settlement, location and time

Location	Region	State	Latitude	Longitude	Dusk	Outdoor	Indoor	
						Dawn	Dusk	Dawn
Granada	Acrelandia	Acre	− 9.752	− 67.071	24	30	19	23
Remansinho	Labrea	Amazon	− 9.497	− 66.582	16	–	16	–

### Genetic diversity of *Nyssorhynchus darlingi* associated with biting behavior

#### Location: indoor vs outdoor

**Fig. 2:**
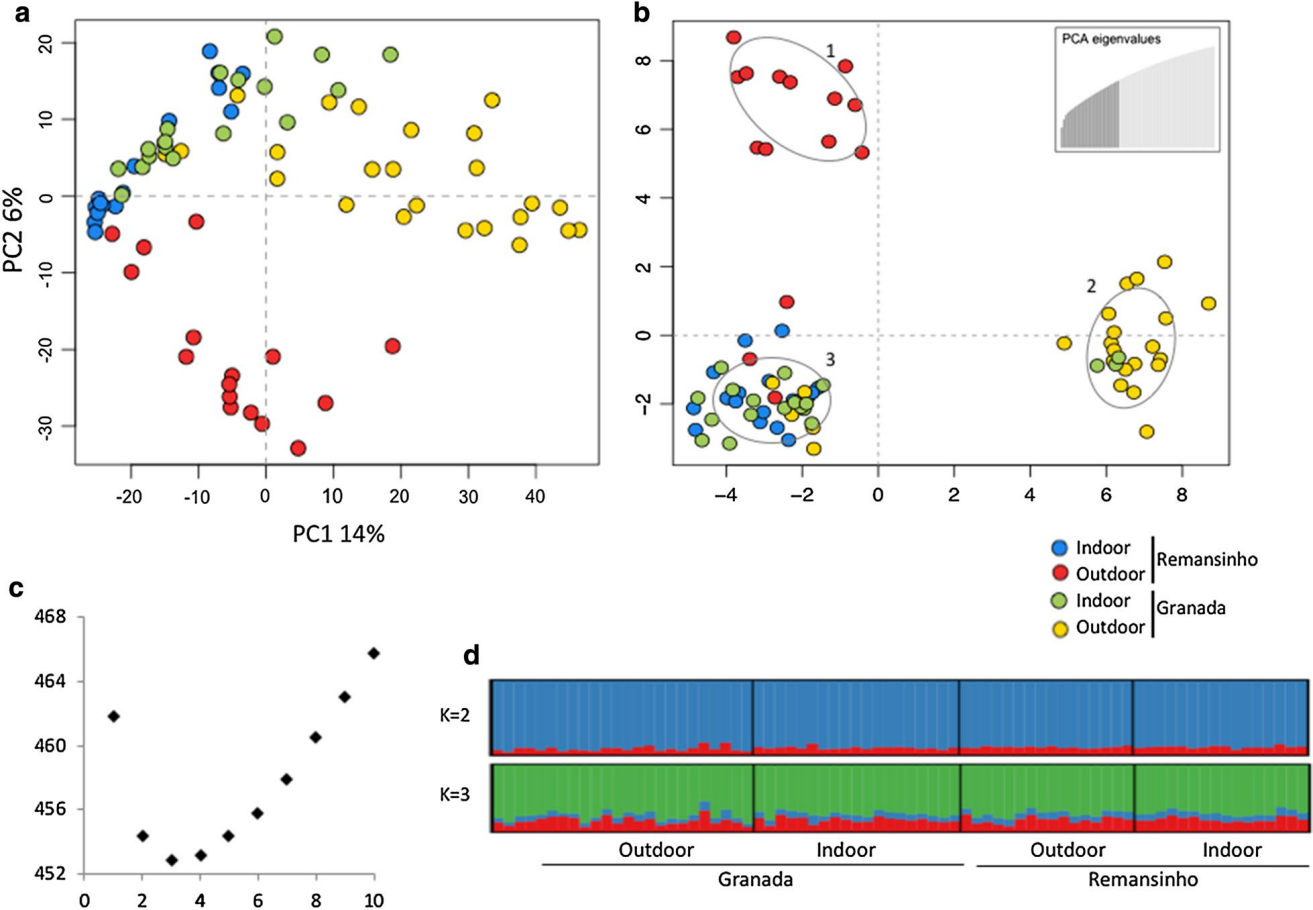
Results of PCA, and clustering by DAPC and STRUCTURE of 944-locus SNP dataset, comparing endophagic and exophagic *Nyssorhynchus darlingi*. **a** PCA of outdoor samples (red and yellow dots) and indoor samples (blue and green dots) from Granada and Remansinho. **b** DAPC ordination of all samples in the three genetic clusters (1–3) in two axes; top right box: number of PCs retained (*n* = 28). **c** Bayesian information criterion (BIC) to estimate the appropriate number of genetic clusters, which is the lowest BIC value. **d** Results of STRUCTURE analysis: each column represents an individual and colors reflect genetic clusters assignment (*K* = 2 and *K* = 3)

Dataset analyses were conducted to test the hypothesis of population structure among indoor and outdoor samples collected at dusk in Granada and Remansinho. After filtering, 944 SNP-loci were genotyped in at least 50% of all individuals from the four groups. STRUCTURE analysis of the SNP dataset supported 2 genetic clusters, and individuals are admixed among the two clusters, suggesting that all individuals belong to a single panmictic population (Fig. 2d). Principal components analysis (PCA) showed that most of indoor specimens were concisely grouped together regardless of the collection location, whereas outdoor individuals were found in two different areas in the plot (Fig. 2a; PC1 = 14%, PC2 = 6%). Discriminant analysis of principal component (DAPC) showed *K* = 3 as the best number of genetic clusters i.e. the lowest Bayesian information criterion (BIC) (Fig. 2c). Cluster “1” had only outdoor *Ny. darlingi* specimens from Granada and cluster “2” had nearly all outdoor individuals from Remansinho, whereas cluster “3” included indoor individuals from both locations (Fig. 2b). Indoor groups showed a higher percentage of polymorphism compared with outdoor ones (Table 2) even when subdivided into dusk and dawn collections (Table 3). Interestingly, the highest pairwise *F*_*ST*_ was between indoor Remansinho and outdoor Granada samples (*F*_*ST*_ = 0.177, *P* < 0.0001), and the lowest was between indoors samples (*F*_*ST*_ = 0.094, *P* < 0.0001) (Table 4).

**Table 2:** Summary statistics for the indoor- and outdoor-collected *Nyssorhynchus darlingi*

Location	Collection	Private	Variant sites	Polymorphic sites	% Polymorphic loci
Granada	Indoor	111	920	469	0.337
	Outdoor	104	937	379	0.267
Remansinho	Indoor	134	938	532	0.375
	Outdoor	96	936	468	0.330

**Table 3:** Summary statistics for the indoor/outdoor- and dusk/dawn-collected *Nyssorhynchus darlingi*

Location	Time	Private	Variant sites	Polymorphic sites	% Polymorphic loci
Indoor	Dusk	55	556	243	0.289
	Dawn^a^	85	557	315	0.374
Outdoor	Dusk	70	556	190	0.226
	Dawn^b^	91	557	291	0.346

^a,b^Specimens included in these categories are only from Granada; none were collected in Remansinho

**Table 4:** Pairwise *F*_*ST*_ between indoor- and outdoor-collected *Nyssorhynchus darlingi*

	Granada Outdoor	Granada Indoor	Remansinho Outdoor
Granada Indoor	0.140		
Remansinho Outdoor	0.146	0.140	
Remansinho Indoor	0.177	0.094	0.129

#### Time: dusk vs dawn

**Fig. 3:**
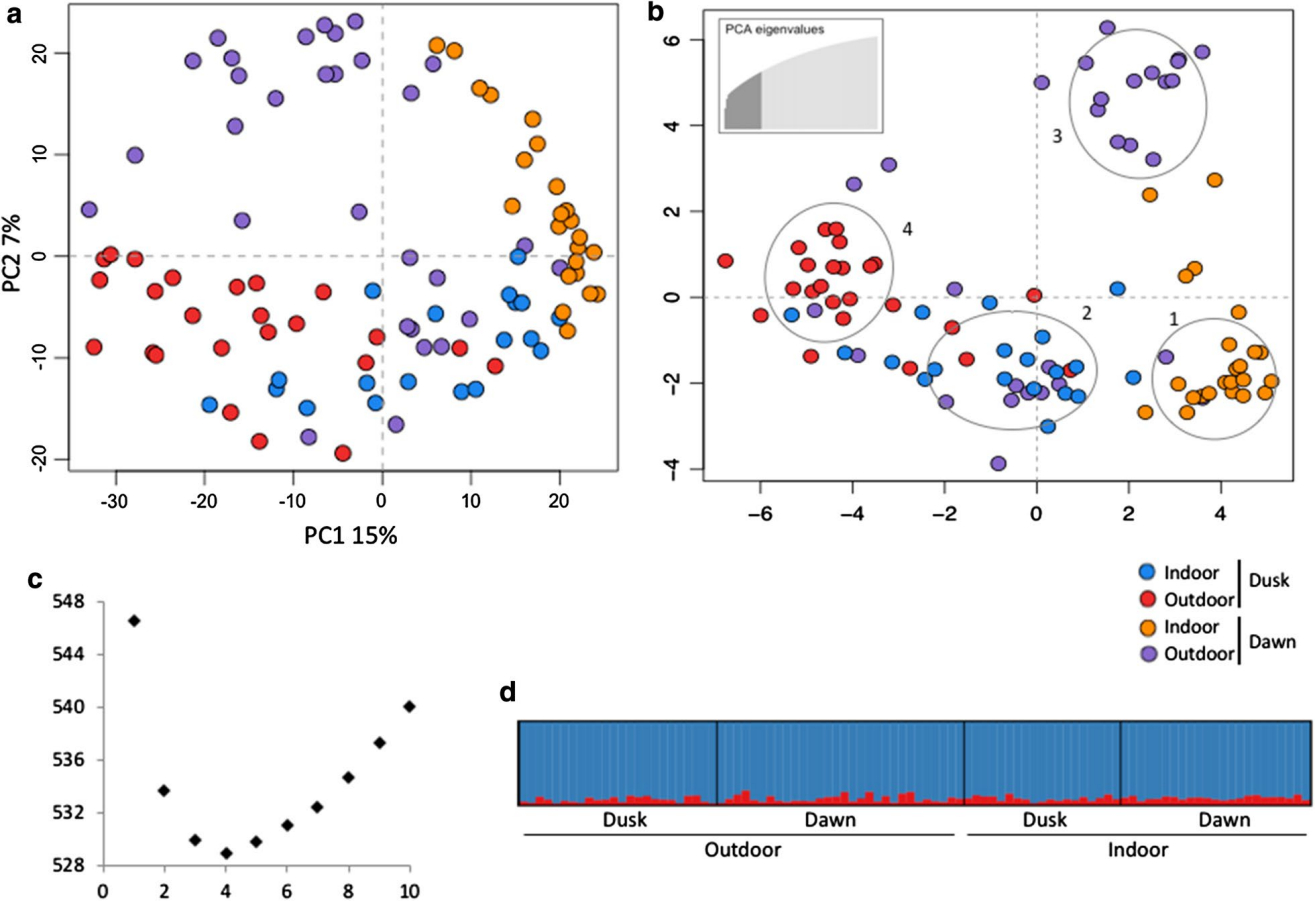
Results of PCA, and clustering by DAPC and STRUCTURE of 589-locus SNP dataset, comparing dusk/dawn endophagic and exophagic *Nyssorhynchus darlingi*. **a** PCA of dusk samples (red and blue dots) and dawn samples (purple and orange dots) from Granada. **b** DAPC ordination of all samples in the four genetic clusters (1–4) in two axes; bottom right box: number of PCs retained (*n* = 23). **c** Bayesian information criterion (BIC) to estimate the appropriate number of genetic clusters, which is the lowest BIC value. **d** STRUCTURE result: each column represents an individual and colors reflect genetic clusters assignment (best *K* = 2)

Four groups of samples from Granada, indoors and outdoors, collected at dusk and dawn, were analyzed in this set to test the hypothesis of *Ny. darlingi* population structure between indoor *vs* outdoor feeding and dawn *vs* dusk feeding. A total of 589 SNP-loci were genotyped in at least 50% of individuals. STRUCTURE result of the SNP dataset supported a lack of population genetic structure, although the optimal number of genetic clusters was 2 (Fig. 3d). All individuals were admixed for the two clusters, indicating that they belong to a single population. The first axis (15%) in the PCA divided indoor and outdoor samples, whereas the second axis (7%) separated time of collection (Fig. 3a). The lowest BIC for *K*-means was 4 in the DAPC where essentially the four groups were partitioned (Fig. 3b, c). Samples collected at dawn showed a higher percentage of polymorphic loci compared with those collected at dusk (Table 5). For this dataset, the highest pairwise *F*_*ST*_ was between outdoor dusk and indoor dawn samples (*F*_*ST*_ = 0.259, *P* < 0.0001), and the lowest was between outdoors samples (*F*_*ST*_ = 0.081, *P* < 0.0001) (Table 5).

**Table 5:** Pairwise *F*_*ST*_ between indoor/outdoor- and dusk/dawn-collected *Nyssorhynchus darlingi*

	Indoor Dawn^a^	Indoor Dusk	Outdoor Dawn^b^
Indoor Dusk	0.155		
Outdoor Dawn^c^	0.134	0.102	
Outdoor Dusk	0.259	0.139	0.081

^a,b,c^For these categories there were no specimens from Remansinho; only from Granada

## Discussion

Heterogenous biting behavior is quite characteristic of *Ny. darlingi* throughout the species’ range in Central and South America [2, 25, 28, 44]. Early studies reported its tendency for indoor blood-feeding behavior, i.e. endophagy (reviewed in [4]), but over time a variety of endo:exophagy and endo:exophily ratios have been reported and correlated with different local environmental variables. One example is the shift towards increased exophagy after a vector control intervention such as long-lasting insecticide nets (LLINs) and/or indoors residual spraying (IRS) described in *Ny. darlingi* [45] and other anophelines [46–48].

Despite numerous entomological studies investigating the different aspects of *Ny. darlingi* behavior, few studies focus on the genetic basis of these phenotypic traits. In fact, this study is the first to detect genetic markers associated with exophagy and endophagy as well as biting times (dusk/dawn) in *Ny. darlingi* females. Previous studies with other species such as *An. gambiae* (*s.l.*) and *An. funestus* showed genetic differentiation regarding divergent behavior using chromosomal inversion karyotypes frequencies [49–51]. Moreover, recently, a genetic component was detected for host choice for blood-feeding in *An. arabiensis* linked to two paracentromeric chromosome inversions [52]. However, there is still a lack of studies at such a fine scale, which may be due to the relatively high cost of whole genome sequencing and the complexity of association mapping studies. To achieve high power in genome-wide association studies prior genomic knowledge is required, i.e. linkage disequilibrium, which the current version of the *Ny. darlingi* genome assembly does not provide. Thus, we are aware of the limitations of the present study in identifying the precise location of the causal genetic variants.

Reduced representation genome-sequencing methods, such as ddRAD, have proven to be powerful tools for the assessment of a large number of SNPs on a genome-wide scale, with considerably lower library construction and sequencing effort [53, 54]. However, these approaches suffer from sampling biases, i.e. allele dropout (ADO), due to the absence or polymorphism within a restriction site [55]. Here, in order to minimize any bias from allele dropout or from other sources, we applied two strict filters for all sets of analysis: no prior information of population and only loci present in more than half of all individuals were selected.

Our results, using 994 genome-wide SNPs, showed a lack of population structuring related to indoor and outdoor biting behavior. On the other hand, PCA and DAPC analysis showed that the same markers are present in indoor mosquitoes collected at both Granada and Remansinho (Fig. 2). Although, overall *F*_*ST*_ values are significant between these groups, and a consistently lower value was detected for endophagic populations, no different clustering assignment were found using STRUCTURE analysis. The single endophagic group could be explained by similar selection of *Ny*. *darlingi* females related to environmental conditions within houses such as temperature, shelter and blood meal availability. In fact, Paaijmans & Thomas [56] showed that indoor environments are warmer than outdoor ones and usually present less daily variation. Besides temperature, indoor residual spraying could be an important driver in the selection of mosquito females. In contrast to the present study, PCA and STRUCTURE analysis of SNPs of *Ny. darlingi* from two localities in Loreto, Peru, Cahuide and Lupuna, detected no genetic differentiation between endophagic and exophagic specimens, nor between specimens collected at dusk *vs* dawn [36]. However, Cahuide and Lupuna in Loreto are both older riverine communities, with similar microsatellite profiles and levels of deforestation [21]. We hypothesize that the contrast and difference in outdoor environments between the two Acre populations of Granada and Remansinho is the main driving force for the genetic diversity detected in the present study.

In terms of the time of biting behavior in *Ny. darlingi,* the present study showed a non-random distribution of individuals in the PCA and clustering in DAPC, besides lack of population structuring. To avoid confounding effects of microgeographical population structure, only one collection point was used in this analysis. As shown in the first set of analysis, PC1 segregated endophagic and exophagic *Ny. darlingi* females (Fig. 3). In addition, PC2 segregated dusk and dawn individuals. In fact, DAPC presented four genetic clusters containing samples from each category. Across its broad distribution, *Ny. darlingi* populations present a range of peak biting times and patterns, i.e. unimodal, bimodal, trimodal [4, 33]. Biting time variation of *Ny. darlingi* has been associated with seasonality [23, 57] and local and ecological factors [58]. Additional sampling could help to determine whether the differentiation detected in this study is consistent between populations of *Ny. darlingi* showing similar behavior patterns.

It is widely accepted that behavioral aspects of anopheline species such as host preference, local and time of biting and resting location after a blood meal have a major impact on malaria transmission dynamics in endemic areas [59, 60]. The apparent discrepancy between the lack of population structuring and the non-aleatory distribution of markers related to behavior in our samples could be due to the lack of association between behavior and mating-related genes. Probably, at the time of host seeking, the female is already inseminated. From an evolutionary point of view, it is tempting to speculate that this behavioural diversity of related genes of *Ny. darlingi* contributed to its dispersion in different niches within the Amazon region. Together with recent events directly attributable to human presence, such as deforestation and land use changes on a massive scale [4, 11, 12] we hypothesize that these processes could select different traits of *Ny. darlingi* and may explain the behavior heterogeneity observed in these different studies. A better understanding of the genetic diversity in *Ny. darlingi* regarding its behavior may help to predict and improve vector control methods and effectiveness of malaria frontline interventions.

## Conclusions

In this study, we provided evidence of genetic diversity associated with biting behavior in the major Neotropical malaria vector *Ny. darlingi* and showed that this association is not due to population structuring. Mosquito behavior is one of the multiple factors directly influencing in the impact of measures of vector control. Molecular tools, such as presented in this study, could help to address appropriate vector surveillance and control on a local-scale perspective. It is emphasized that additional ecological and genomic studies may help to understand the genetic basis of *Ny. darlingi* behavior by the identification of relevant genes and/or genomic regions.

## Additional file

**Additional file 1.** Per-individual *An. darlingi* detail of the number of sequence reads and unique stacks genotyped.

## Data Availability

Data supporting the conclusions of this article are included within the article. The dataset analyzed are available in the NCBI SRA BioProject PRJNA29824, BioSample: SAMN04202372–SAMN04202499.

## Abbreviations

BIC: Bayesian information criterion
DAPC: discriminant analysis of principal components
ddRADseq: double digestion restriction-site associated DNA sequencing
PCA: principal components analysis
SNP: single nucleotide polymorphism
